# Food-drug interactions: Knowledge among pharmacists in Jordan

**DOI:** 10.1371/journal.pone.0234779

**Published:** 2020-06-17

**Authors:** Mohammed Zawiah, Al-Motassem Yousef, Amer Hayat Khan, Fahmi Y. AL-Ashwal, Amal Matar, Batool ALKhawaldeh, Rand Nassar, Rami Abduljabbar, Abdullah Abdulmajid Abdo Ahmed

**Affiliations:** 1 Discipline of Clinical Pharmacy, School of Pharmaceutical Sciences, University Sains Malaysia, Penang, Malaysia; 2 Department of Biopharmaceutics and Clinical Pharmacy, School of Pharmacy, The University of Jordan, Amman, Jordan; 3 College of Pharmacy, Yemeni University of Sciences and Technology, Taizz, Yemen; 4 Department of Pharmacy Practice, Kulliyyah of Pharmacy, International Islamic University, Malaysia; College of Pharmacy & Health Sciences, UNITED STATES

## Abstract

**Background:**

Pharmacists have crucial role in providing drug information and medication counseling to patients. This survey aimed to benchmark the current knowledge of the pharmacists concerning food-drug interactions (FDIs) in Jordan.

**Methods:**

A cross-sectional study was conducted in Amman, the capital and largest city of Jordan, using a validated questionnaire. It was distributed to pharmacists working in community and hospital pharmacies using a convenience sampling technique. Descriptive and inferential statistics were performed in this study.

**Results:**

A total of 340 questionnaires distributed, 300 (88%) pharmacists responded. Over 50% of pharmacists claimed that they have sufficient knowledge regarding FDI. Virtually, the overall median (interquartile range) knowledge score was 18 (15–21), approximately 60%. The highest knowledge scores were for alcohol-drug interactions section (66.6%) followed by both common food-drug interactions and the timing of drug intake to food consumption sections with a score of (58.3%) for each, reflecting a suboptimal knowledge of FDIs among the pharmacists.

**Conclusion:**

Pharmacists had unsatisfactory knowledge about common FDIs, with no significant difference between hospital and community pharmacists. Therefore, more attention and efforts should be played to improve awareness about potential food-drug interactions.

## Introduction

Food and drugs are essential components of the patient’s therapeutic plan. Like drugs, food has increasingly become a key element in the prevention and treatment of diseases [1]. However, the interaction between them is one of the main challenges, especially for oral medications [2]. Food-drug interactions (FDIs) are defined as alterations in drugs’ pharmacokinetics or pharmacodynamics as a result of food, or changes to nutrients caused by drugs [3]. In this light, FDIs may enhance or inhibit the absorption, distribution, metabolism, and excretion of drugs or alter their clinical or physiological effects on the body [4, 5]. Few studies have reported the prevalence of FDIs in clinical practice [6–9]. The prevalence ranged from 6.3% for intensive care unit patients with enteral nutrition [7] to 58.5% among elderly patients [9].

The interaction between food and drugs could lead to treatment failure or predispose the patients for many side effects that could be life-threatening [3]. For instance, the interaction between tyramine, which is a substrate found in aged and fermented foods such as cheese, and monoamine oxidase inhibitors (MAOIs) may lead to a hypertensive crisis and myocardial infarction [10, 11]. Some groups of patients have a higher risk for FDIs and they are more prone to serious side effects such as elderly patients, hospitalized patients, patients on drugs with narrow or low therapeutic index and patients with chronic diseases [12]. Therefore, health professional’s knowledge about potential FDIs is essential, and being able to identify individuals with high risk is required for safe and effective use of medications.

One of the standards that were established by the joint commission on the accreditation of healthcare organizations is the requirement that pharmacists should take thorough medication history for patients during hospital admission, and they should be aware of clinically relevant drug-drug interactions and FDIs and to provide adequate counseling for patients about potential FDIs before discharge [13]. Pharmacists contribute greatly to the safe and effective use of medications, and they are usually the first healthcare professionals approached by patients for medical advice [14]. Pharmacists are not only responsible for drug dispensing but also for reviewing medications and, more importantly, counseling the patients. Thus, pharmacists’ knowledge of FDIs is essential for proper patients’ education, avoiding severe side effects, and improving treatment efficacy. Few studies have evaluated healthcare professionals’ knowledge of FDIs [15–19]. However, no research has been done in Jordan. Therefore, our aim in this survey was to assess pharmacists’ knowledge of common food-drug interactions. Also, to evaluate the factors that are commonly associated with adequate knowledge among the study population. Moreover, to see if there is a significant difference in knowledge between the community and hospital pharmacists.

## Methods

### Design and setting of the study

A cross-sectional study was conducted over a period of three months from January to March 2019 among the community and hospital pharmacists in Amman, Jordan. A validated structured questionnaire was distributed using a convenience sampling technique. Respondents filled out the questionnaire in the presence of a researcher to clarify the questions and to make sure that respondents’ answers were based on their current knowledge. Independent and chain pharmacies, as well as public and private hospitals, were covered. Ethical approval was obtained from the institutional review board at Jordan University. Written informed consent was given for respondents who agreed to participate in the study.

### Study tool and scoring system

This survey was conducted using a revised questionnaire from previous published studies [15, 19]. The questionnaire was first checked by experts and then tested with a pilot study on a convenient sample of 10 pharmacists to ensure the content and face validity of the questionnaire. The final form is consisting of four main sections. Section one included the socio-demographic data of participants, such as age, gender, qualifications, workplace, and experience, in addition to three general questions related to pharmacists' knowledge of FDIs (“Do you think you have enough information about food-drug interactions?”, “Which of the following age-groups is most susceptible to food-drug interactions?”, and “What is the main source for your knowledge of Food-drug interactions). The second section contained twelve closed-ended (yes/no/I don’t know) questions to evaluate the general knowledge of the participants about the common food-drug interactions. In this section, we included common food-drug interactions that have been cited in previous similar studies, some of these interactions could result in serious side effects (Grapefruit with atorvastatin, amiodarone and some antibiotics (Example: Erythromycin), MAOI with cheese and fermented food, spironolactone with potassium rich foods, coumadin with green vegetables, theophylline with excessive coffee and tea), others could result in decreasing the efficacy of medication to a huge extent especially when the food is consumed regularly, in high amount or with a narrow therapeutic medication (Tetracycline with milk and dairy products, digoxin with wheat bran, levodopa with protein-rich food, levothyroxine and cauliflower, diazepam and caffeine). Section three included another twelve closed-ended questions to assess the knowledge of participants regarding the appropriate timing of medications intake with respect to food. The last section contained six questions exploring the knowledge of pharmacists towards drug-alcohol interactions.

A score of one was given for each correct answer, and zero was given for the incorrect or 'I do not know' ones. The total score for each respondent was the summation of the scores in each section, while the overall knowledge score was the summation of all sections scores, which ranged from zero to thirty, and was subcategorized into three levels: Excellent knowledge ≥ 80% (answered ≥ 24 correct answers), moderate knowledge 50%—< 80% (15–23 correct answers), and poor knowledge < 50% (< 15 correct answers).

### Statistical analysis

We applied both descriptive and inferential statistics using IBM statistical package for the social sciences (IBM SPSS®21 software IBM, Armonk, New York, USA). Categorical variables were assessed using frequencies and percentages, while the continuous variables were reported as median and interquartile range (IQR). Associations between the overall knowledge scores and the collected sample covariates (specialty: hospital vs. community pharmacists; gender: males vs. females; age: ≤33 years vs. >33 years; experience; < 10 years vs. ≥ 10 years, university type; public vs. private and employment status; owner vs. employee) were examined using Mann-Whitney test, and the Kruskal–Wallis test was used to assess the association between the overall knowledge scores and the highest degree obtained by respondents. All results with p < 0.05 were considered statistically significant. The internal consistency for the questionnaire was assessed using Cronbach’s α test, and the result was (0.73).

Sample size was calculated using Raosoft® online calculator (www.raosoft.com). Based on the latest statistics reported by Jordan Pharmacists Association, there were approximately 1568 pharmacies in Amman at the time the study was conducted. An estimated 309 participants were required, to achieve 95% confidence interval level and accepted margin of error of 5% and considering a 50% response distribution.

## Results

### Respondent demographics

Of 340 questionnaires distributed, 300 (88%) pharmacists responded, of whom 90 (30%) were male, and 210 (70%) were female. Of these respondents, 55.3% worked in the community pharmacies, while 44.7% of them were in the hospital pharmacies. The median (IQR) for age and experience was 30.5 (26–39) and 6 (3–14) years, respectively. The respondents’ characteristics are shown in Table 1.

**Table 1:** The socio-demographic characteristics of study sample (n = 300).

Characteristics	Frequency (Percentage)
Gender	
Male	90 (30.0)
Female	210 (70.0)
Age, years	
≤ 31	162 (54.0)
>31	138 (46.0)
Median (IQR)	30.5 (26–39)
Experience, years	
< 6	130 (43.3)
≥ 6	170 (56.7)
Median (IQR)	6 (3–14)
Area of work	
Community pharmacy	166 (55.3)
Hospital pharmacy	134 (44.7)
Highest degree obtained	
Bachelor of pharmacy	244 (81.3)
Doctor of pharmacy	48 (16.0)
Master	8 (2.7)
University type	
Public	195 (65)
Private	99 (33)
Missing	6 (2)
Employment status	
Employee	263 (87.7)
Owner	37 (12.3)

IQR = Interquartile range

### Pharmacists' knowledge regarding food-drug interactions

Pharmacists were initially asked about the main source for their knowledge of food-drug interactions and which age-category is more prone to FDIs. Two-thirds of respondents (66.3%) stated that university education was their primary source for FDIs knowledge. Also, a large proportion (64.6%) of participants reported that older people are more susceptible to food-drug interactions than other age-groups. Furthermore, over 50% of participants claimed that they have sufficient knowledge regarding food-drug interactions.

In this survey, pharmacists demonstrated a high knowledge score for some individual questions regarding food-drug interactions. For instance, the knowledge score for the interaction between tetracycline with milk and dairy products, warfarin with green vegetables and atorvastatin with grapefruit, were (87.3%), (70.3%), and (79.7%), respectively. Moreover, approximately two-thirds of participants answered correctly about the interaction of antibiotics with grapefruit juice (65.3%), and the interaction of theophylline with excessive coffee and tea (66%).

On the other hand, less than half of pharmacists were aware of the interactions between digoxin with wheat bran, diazepam with caffeine, and levothyroxine with cauliflower with knowledge scores of 49%, 44.7% and 41.7, respectively. Also, over 53% of pharmacists were able to identify the interaction of spironolactone with potassium-rich sources (n = 160) as well as the interaction of levodopa with protein-rich food (n = 160) (Table 2). The median (IQR) knowledge score for this section was 7 (6–9). No significant difference was found in knowledge between community and hospital pharmacists.

**Table 2:** Knowledge assessment for pharmacists about food-drug interactions (n = 300).

Questions	Correct answers, N (%)
Amiodarone with grapefruit	179 (59.7)
Atorvastatin with grapefruit	211 (70.3)
Levothyroxine with cauliflower	125 (41.7)
Diazepam with caffeine	134 (44.7)
Coumadin with green vegetables	239 (79.7)
Theophylline with excessive coffee and tea	196 (65.3)
Tetracycline with milk and dairy products	262 (87.3)
MAOI with cheese and fermented food	204 (68.0)
Digoxin with wheat bran	147 (49.0)
Levodopa with protein-rich food	161 (53.7)
Antibiotics with grapefruit juice	198 (66.0)
Spironolactone with potassium rich foods	160 (53.3)

### Drug to food time interval

For this section, the timing of omeprazole intake with regards to food was correctly identified by the vast majority of pharmacists (94.3%), followed by levothyroxine (78%) and metformin (71.3%). On the other hand, poor knowledge about timing (average <50%) was apparent for 5 out of the 12 medications, which were carbamazepine, methotrexate, propranolol, calcium carbonate supplements, and erythromycin stearate with knowledge percentage of 41.7%, 39%, 39.3%, 35.7%, and 35.3% respectively. Thus, the median (IQR) knowledge score for all medications in this part was 7 (5–8). (Table 3). Similarly, no significant difference was found in knowledge between community and hospital pharmacists.

**Table 3:** Pharmacists’ knowledge about timing of drug intake with respect to food (n = 300).

Drug	Correct answers, N (%)
Carbamazepine	125 (41.7)
Methotrexate	117 (39)
Isotretinoin	204 (68)
Omeprazole	283 (94.3)
Glipizide	171 (57)
NSAIDs	172 (57.3)
Levothyroxine	234 (78)
Griseofulvin	154 (51.3)
Metformin	214 (71.3)
Calcium carbonate supplement	107 (35.7)
Erythromycin stearate	106 (35.3)
Propranolol	118 (39.3)

### Alcohol-drugs interactions

**Fig 1:**
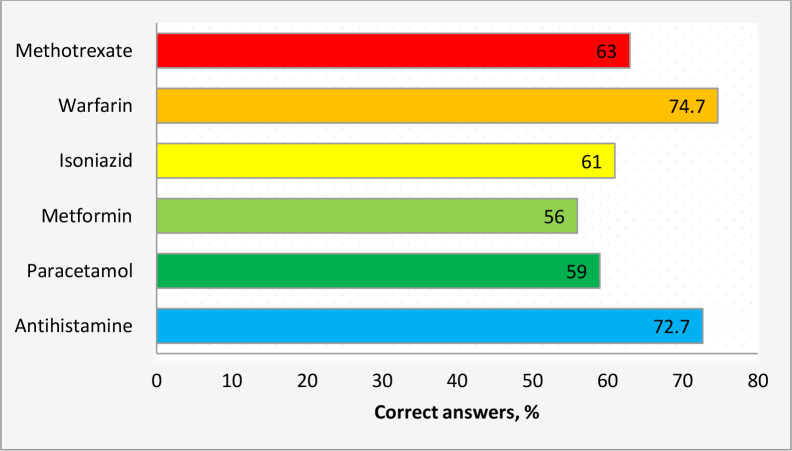
Participants' knowledge with regards to alcohol-drugs interactions.

With regards to alcohol-drugs interactions items, the median (IQR) score was 4 (3–6). In terms of the individual questions, warfarin and antihistamines received the highest percentages of (74,7%) and (72,7%), respectively, followed by methotrexate (63%), isoniazid (61%), paracetamol (59%), with metformin being the lowest (56%), Fig 1.

Generally speaking, only few of pharmacists (7%) achieved an excellent knowledge level, and the overall median (IQR) knowledge score was 18 (15–21) out of 30 which is insufficient. Regarding the association between socio-demographic factors and overall pharmacists’ knowledge score, we found that all factors have no significant effect on the knowledge score of pharmacists except for university type and alcohol-drug interaction overall score (Table 4).

**Table 4:** Influence of respondents’ characteristics on their knowledge level about food-drug interactions, timing of taking medications and alcohol-drug interactions (n = 300).

Variable	Median knowledge score of FDIs (IQR)	*P value	Median knowledge score of timing for taking drug (IQR)	*P value	Median knowledge score of alcohol-drug interaction (IQR)	*P value	Overall median knowledge score (IQR)	* P value
Gender								
Male	7 (5–9)	0.784	7 (5–8)	0.232	4 (3–6)	0.292	18 (14–21)	0.213
Female	7 (6–9)		7 (5–8)		4 (3–6)		18 (15–21)	
Age group								
≤ 31	8 (6–9)	0.281	7 (6–8)	0.523	4 (3–6)	0.974	18 (15–22)	0.385
> 31	7 (5.75–9)		7 (5–8)		4 (3–6)		17 (15–21)	
Experience (years)								
< 6	8 (6–9)	0.122	7 (6–8)	0.377	4 (3–6)	0.462	19 (16–21)	0.080
≥ 6	7 (5–9)		7 (5–8)		4 (3–6)		17 (15–21)	
Highest degree obtained								
Bachelor of pharmacy	8 (6–9)	0.493	7 (5–8)	0.992	4 (3–6)	0.670	18 (15–21)	0.737
Doctor of pharmacy	7 (6–9)		7 (5–8)		4 (3–6)		18 (15–20)	
Master	6 (4–9)		7 (6–7)		3.5 (2.25–4.75)		16 (14.25–21.5)	
University type								
Public	7 (5–9)	0.105	7 (6–8)	0.584	4 (3–6)	0.018	17 (15–21)	0.058
Private	8 (6–9)		7 (5–8)		5 (3–6)		19 (16–21)	
Area of work								
Community pharmacy	8 (5–10)	0.127	7 (5–8)	0.318	4 (3–6)	0.834	18 (15–22)	0.823
Hospital pharmacy	7 (6–9)		7 (5–8)		4 (3–6)		18 (15–21)	
Employment status								
Employee	7 (6–9)	0.585	7 (6–8)	0.110	4 (3–6)	0.180	18 (15–21)	0.302
Owner	8 (5–10)		6 (4–8)		3 (1.5–5.5)		18 (14–20)	

*Mann-Whitney test, and the Kruskal–Wallis were used. IQR = Interquartile range.

## Discussion

This is the first study of its kind in Jordan to assess the level of pharmacists’ knowledge towards FDIs. The present findings contribute to the limited literature available in the middle-eastern countries about pharmacists’ knowledge of FDIs and allow for future comparative work and research world widely. Also, the results could be utilized by educational and healthcare policy-makers in designing appropriate educational interventions to promote the knowledge of clinically significant FDIs among pharmacists and other healthcare professionals.

In the present study, the overall median knowledge score (IQR) was 18 (15–21), approximately 60%. The highest knowledge scores were for alcohol-drug interactions section (66.6%) followed by both common food-drug interactions and the timing of drug intake to food consumption sections with score of (58.3%) for each, reflecting a suboptimal knowledge of FDIs among the pharmacists. These findings were consistent with previous studies that reported a knowledge gap of FDIs among pharmacists and other healthcare professionals [15, 19, 20]. In this light, A recent Palestinian study revealed inadequate knowledge about FDIs among pharmacists, their overall knowledge score was reported to be 17.9 out of 29, approximately 61.7% [19]. Similarly, in a cross-sectional study done in India among 200 doctors (Professors, Post-Graduates (PGs) and Interns). The knowledge gap was higher for Interns and PGs than that of Professors. The mean knowledge scores were 21.35 ± 4.2, 22.89 ± 3.72, 26 ± 4.08 for interns, PGs, and professors, respectively, with 31 being the maximum score [15].

For the common FDIs, pharmacists demonstrated adequate knowledge for some interactions, mainly tetracycline with milk and dairy products, and warfarin with green vegetables with scores of 87.3% and 79.7%, respectively. This adequate knowledge for these interactions could be due to their early discovery, use, and being extensively researched [21], making them famous examples of FDIs in literature, university education, and practice. Milk and dairy products contain a high amount of calcium and magnesium that binds to tetracycline forming insoluble complexes, preventing their absorption, and eventually resulting in low bioavailability [22]. For warfarin, high consumption of vitamin K-rich sources such as broccoli, Brussels sprouts, and Lettuce will increase the production of clotting factors, diminishing the therapeutic effect of warfarin, predisposing the patients for clots [23].

Knowledge about grapefruit-drug interactions was assessed with three commonly used medications: Amiodarone, atorvastatin, and antibiotics. More than eighty-five medications are known or predicted to interact with grapefruit, some of which can greatly affect the efficacy and safety [24]. Participants’ knowledge scores ranged from 59.7% for amiodarone to 70.3% for atorvastatin. This knowledge score among the pharmacists is not sufficient, especially when the predicted interaction risk of serious side effects for both medications is rated as high, with the risk of rhabdomyolysis for atorvastatin and torsade de pointes (TdP) for amiodarone [25]. Cases of rhabdomyolysis as a result of grapefruit-statins interaction have been reported [26]. For the interaction between grapefruit and antibiotics, approximately one-third of participants wrongly believe that grapefruit juice can be consumed safely with all antibiotics. In this light, concomitant use of erythromycin with grapefruit juice is rated as high predicted interaction risk for torsade de pointes, a life-threatening arrhythmia [24].

Accordingly, the food and drug administration (FDA) has required that prescriptions should include a warning against drinking grapefruit with these medications [27]. Similarly, a significant proportion (68%) of respondents provided correct answers for the knowledge question of MAOIs interaction with cheese and fermented food. Aged cheese and other fermented food contain tyramine, which a sympathomimetic-like agent. This interaction could result in a hypertensive crisis and myocardial infarction [10, 11]. Health professionals’ awareness about such interactions and counseling the patients about them could prevent life-threating events.

On the other hand, more than half of the participants were not aware of the interaction of levothyroxine with cauliflower, and diazepam with caffeine. Coadministration of levothyroxine, which is a narrow therapeutic medication, with a large amount of cauliflower can interfere with iodine intake and thyroid function resulting in goiter [28]. This maybe of special concern for patients in Jordan, where cauliflower occupies an important place in the Jordanian cuisine [29]. For diazepam, coadministration with caffeine-containing beverages and foods may result in a reduction in its sedative and anxiolytic effects [30]. Diazepam is known to have calm, sedating and anxiolytic effects. In contrast, caffeine is mainly taken as a stimulant and could have anxiogenic effect when taken in high amount, especially among patients with preexisting anxiety disorders [30, 31]. Caffeine is increasingly consumed in many countries [32]. Therefore, pharmacists’ knowledge of such interactions is important to optimize therapy.

Almost half of the participants answered incorrectly the question related to the interaction between digoxin, which is a low therapeutic index agent, and wheat bran, which is high in fibers. The intake of both agents concomitantly may reduce digoxin absorption by 16% [33]. Levodopa interaction with protein-rich food is another important interaction. Only 53.7% of pharmacists were aware of it. Concomitant intake of Levodopa with high protein diets can cause a reduction in its absorption, leading to motor fluctuations and therapeutic effects reduction in patients with Parkinson’s disease [34]. Similarly, approximately the same proportion of participants (53.3%) were aware of the interaction between spironolactone and potassium-rich sources. Spironolactone is known as a potassium-sparing diuretic. Consumption of a diet rich in potassium with spironolactone can elevate potassium plasma levels, leading in severe cases to potentially fatal hyperkalemia [33, 35]. Therefore, inadequate pharmacists’ knowledge of common FDIs leads to improper patients’ education, and this could result in harmful consequences.

Regarding alcohol-drug interactions, a significant proportion of pharmacists were familiar with the interaction of alcohol with warfarin and antihistamines and, to a lesser extent, for the interaction with methotrexate, isoniazid, paracetamol, and metformin. Taking these medications concomitantly with alcohol could lead to undesirable outcomes. In this regard, the consumption of alcohol with warfarin increases the patient’s risk of bleeding [36]. Also, using alcohol concurrently with methotrexate, isoniazid, and paracetamol enhances the hepatotoxic effect for these medications [37]. Furthermore, alcohol intake with antihistamines potentiates their sedative effects and CNS depression [38]. This effect, in turn, increases patient’s risk of falling and accidents [39].

For the drug-to-food time interval, the knowledge score for all medications was 58.3%, indicating unsatisfactory knowledge of medications’ timing with respect to food among pharmacists. However, the majority of pharmacists were conversant about the timing of omeprazole intake with regards to food. This finding is in line with previous studies that demonstrated adequate knowledge for the omeprazole-food time interval among health professionals [15, 18, 19]. This high knowledge score for omeprazole could be attributed by being one of the proton pump inhibitors, which are the most commonly prescribed medications for gastroesophageal reflux disease, stomach and duodenal ulcers in the world [40]. Similarly, a significant proportion of participants (78%) were aware of the time interval between levothyroxine and food, a higher percentage than that reported in Palestine (44.8%) [19]. Levothyroxine should be taken on an empty stomach, usually 30–60 minutes before breakfast, to avoid erratic drug absorption [41]. On the other side, the lowest percentage knowledge scores were for calcium carbonate supplements (35.7%) and erythromycin stearate (35.3%). The presence of different chemical forms of calcium (such as calcium carbonate, calcium citrate) and erythromycin (enteric-coated erythromycin base, erythromycin stearate, and erythromycin ethylsuccinate) with different drug-to-food timing recommendations [42, 43], which may confuse the pharmacists and have contributed to the low level of knowledge for these FDIs.

In the present study, pharmacists’ knowledge was not associated with age, gender, years of experience, degree level, and employment status. This was inconsistent with previous studies. For example, previous reports have shown that age, working experience, and employment status influence the healthcare professionals’ knowledge of FDIs [15, 16, 19]. Similarly, there was no significant difference in the overall knowledge score between community pharmacists and hospital pharmacists. Food drug interaction is not limited to prescription drugs; it can also occur with over-the-counter drugs [44]; that’s why a good knowledge of common FDIs is paramount for both community and hospital pharmacists.

Good pharmacists’ knowledge is a prerequisite for good practice [45]. Thus, pharmacists should be aware of the common FDIs to counsel the patients appropriately. Unfortunately, in Jordan, there are no FDIs focused-courses in the undergraduate or graduate curricula to provide adequate knowledge about potential FDIs. Moreover, training or educational programs for pharmacists and other healthcare professionals about clinically relevant food-drug interactions is lacking. Furthermore, currently, there is no continuing medical education (CME) credits or educational activities for license renewal [14]. The presence of such activities and requirements in Jordan can help in improving the pharmacists’ knowledge and practice about FDIs. For instance, a recent study showed that the majority of nurses working in pediatric out-patients’ clinics improved their knowledge and practices significantly following an educational program of FDIs [17]. Moreover, the frequency of FDIs decreased significantly after a training course that was given for nurses by a clinical pharmacist [46]. Therefore, introducing an obligatory FDIs course for pharmacy students, and implementing continuing education programs and in-service training of clinically significant FDIs for pharmacists working at the hospitals and community pharmacies are recommended to improve the pharmacists’ knowledge of FDIs.

### Limitation of the study

Data was collected mainly from Amman, the capital of Jordan, and convenience sampling method was used. This limits the generalizability of results. Also, this survey assessed the knowledge for only community and hospital pharmacists. Clinical pharmacists and other healthcare professionals were not included. Therefore, further research with a larger sample size, covering more cities, using random sampling method and including other health professionals with educational intervention is recommended.

## Conclusions

In conclusion, pharmacists had unsatisfactory knowledge about common food-drug interactions, with no significant difference between hospital and community pharmacists. Therefore, more attention and efforts should be played to improve awareness about potential food-drug interactions.

## Supporting information

S1 Questionnaire (DOCX)
