# Supplementary material for: Food-drug interactions: Knowledge among pharmacists in Jordan
Source: PLoS One. 2020 Jun 17;15(6):e0234779. doi: 10.1371/journal.pone.0234779 (PMC7299397; doi:10.1371/journal.pone.0234779)
Supplement: S1 Questionnaire — (DOCX) [file pone.0234779.s001.docx]

**Food-drug interactions: Knowledge among pharmacists in Jordan**

**(Questionnaire)**

**Part (1). Demographics and general questions:**

| **- Age**: ...................................... |
| --- |
| **- Gender**: |
| Male Female |
| **- Education level:** |
| Bachelor of pharmacy Doctor of pharmacy Master |
| **- Name of the university (For your last degree):** ..................................  **- Area of work now:**  Community pharmacy Hospital pharmacy  **- Number of experience years:** ................................. |
| **- Employment status:** |
| Employee Owner |
| **- Do you think you have enough information about food-drugs interactions?** |
| Yes No Not sure |
| **-** **Which of the following age groups is most susceptible to food-drug interactions?** |
| Children Adults Elderly |

- **What is the main source for your knowledge of food-drug interactions?**

…………………………….

**Part (2). Drugs-Food Interactions knowledge**

| Can amiodarone be taken with grapefruit? | 1. |
| --- | --- |
| Yes No I don’t know |  |
| Can atorvastatin be taken with grapefruit? | 2. |
| Yes No I don’t know |  |
| Does cauliflower consumption affect the efficacy of levothyroxine? | 3. |
| Yes No I don’t know |  |
| Does caffeine consumption affect the efficacy of diazepam? | 4. |
| Yes No I don’t know |  |
| Patients can eat more leafy green vegetables with Coumadin (warfarin): | 5. |
| Yes No I don’t know |  |
| Patient taking theophylline should avoid excessive coffee and tea: | 6. |
| Yes No I don’t know |  |
| Does milk affect the efficacy of tetracycline? | 7. |
| Yes No I don’t know |  |
| Patients taking monoamine oxidase inhibitors (MAOIs) should avoid eating aged cheeses: | 8. |
| Yes No I don’t know |  |
| Does wheat bran diet affect the efficacy of digoxin? | 9. |
| Yes No I don’t know |  |
| Does protein-rich foods affect the efficacy of levodopa? | 10. |
| Yes No I don’t know |  |
| Grapefruit juice can be safely consumed with all antibiotics: | 11. |
| Yes No I don’t know |  |
| Patients should avoid taking spironolactone with food rich in potassium? | 12. |
| Yes No I don’t know |  |

**Part (3). Knowledge about timing of drug intake with respect to food:**

Please choose the best time to take each medication with respect to food

| Can be taken without regard to food | Two hours after meal | With meal | Before meal with 1/2 hour | Medication |  |
| --- | --- | --- | --- | --- | --- |
|  |  |  |  | Carbamazepine | 13. |
|  |  |  |  | Methotrexate | 14. |
|  |  |  |  | Isotretinoin | 15. |
|  |  |  |  | Omeprazole | 16. |
|  |  |  |  | Glipizide | 17. |
|  |  |  |  | NSAIDs | 18. |
|  |  |  |  | Levothyroxine | 19. |
|  |  |  |  | Griseofulvin | 20. |
|  |  |  |  | Metformin | 21. |
|  |  |  |  | Calcium carbonate supplement | 22. |
|  |  |  |  | Erythromycin stearate | 23. |
|  |  |  |  | Propranolol | 24. |

**Part (4). Knowledge about Drugs-alcohol interactions:**

For each medication, please choose whether there is drug-alcohol interaction or not (“Yes” means **there is** drug-alcohol interaction, “No” means **there is no** drug-alcohol interaction, “I don’t know” means you do not know the answer)

| I don’t know | NO | Yes | Medication |  |
| --- | --- | --- | --- | --- |
|  |  |  | Antihistamine | 25. |
|  |  |  | Paracetamol | 26. |
|  |  |  | Metformin | 27. |
|  |  |  | Isoniazid | 28. |
|  |  |  | Warfarin | 29. |
|  |  |  | Methotrexate | 30. |

Thank you
